# Proof of concept of a workflow methodology for the creation of basic canine head anatomy veterinary education tool using augmented reality

**DOI:** 10.1371/journal.pone.0195866

**Published:** 2018-04-26

**Authors:** Roxie Christ, Julien Guevar, Matthieu Poyade, Paul M. Rea

**Affiliations:** 1 Anatomy Facility, Thomson Building, School of Life Sciences, College of Medical, Veterinary and Life Sciences, University of Glasgow, Glasgow, United Kingdom; 2 School of Simulation and Visualisation, The Glasgow School of Art, Glasgow, United Kingdom; 3 Department of Clinical Sciences, College of Veterinary Medicine, North Carolina State University, Raleigh, NC, United States of America; University of Bari, ITALY

## Abstract

Neuroanatomy can be challenging to both teach and learn within the undergraduate veterinary medicine and surgery curriculum. Traditional techniques have been used for many years, but there has now been a progression to move towards alternative digital models and interactive 3D models to engage the learner. However, digital innovations in the curriculum have typically involved the medical curriculum rather than the veterinary curriculum. Therefore, we aimed to create a simple workflow methodology to highlight the simplicity there is in creating a mobile augmented reality application of basic canine head anatomy. Using canine CT and MRI scans and widely available software programs, we demonstrate how to create an interactive model of head anatomy. This was applied to augmented reality for a popular Android mobile device to demonstrate the user-friendly interface. Here we present the processes, challenges and resolutions for the creation of a highly accurate, data based anatomical model that could potentially be used in the veterinary curriculum. This proof of concept study provides an excellent framework for the creation of augmented reality training products for veterinary education. The lack of similar resources within this field provides the ideal platform to extend this into other areas of veterinary education and beyond.

## Introduction

The practice of medicine and veterinary medicine relies heavily on clinicians thorough and working knowledge of 3D anatomy. Skills needed in a clinician’s repertoire include physical examination, interpretation of imaging data, including advanced imaging, correctly diagnosing, and procedures such as surgery all requiring an in-depth anatomy knowledge [1].

Traditionally, this knowledge acquisition has been a mainstay of medical education and clinical training. Students in the past have relied on lengthy didactic lectures, cadaveric dissection, textbook figures and simplified models to develop their knowledge and anatomy skills [1,2]. Indeed, anatomy has been viewed as the foundation of medical training and budgets have, in the past, been funding dissection programs [1,3]. However, over recent years, there has been a reduction in the amount of time allocated to anatomy teaching, including dissection, and a lack of qualified staff to teach clinically applied anatomy [4–6].

As a consequence of this, and the rapidly progressing field of digital products in anatomical and medical education, there has been an explosion onto the market of a wide range of products [7–10]. Ever since the development of the first major game-changer in digital anatomy of the Visible Human Project, there are now a plethora of tools available [11].

However, unlike human anatomy and medical training using digital products, there has been a serious lack of progress in this field from the veterinary perspective. Certainly, there have been some attempts to develop educational and training materials for the veterinary community. These have however been around isolated cases including the rat brain, frog and limbs of the horse [12–14]. In addition, they did not have sufficient levels of detail that would be required for veterinary students to embed into the curriculum effectively. A more accurate representation was based on the Visible Animal Project (VAP), which attempted to create a 3D database of anatomical items of the dog trunk [15]. However, it lacked the detail, as so many do, of the cranial anatomy of the dog.

Similarly, there has been a rise in the popularity of virtual reality (VR) in human anatomy education [16]. However the challenge here is to make the invisible visible. Even the most skilful dissection of specimens can only reveal certain aspects of structural relationships, and only after significant investment of time and resources [17–19]. VR, however, can make many aspects of the invisible visible to a user in an immersive way, as quickly as navigating to a website or accessing a mobile application. If the VR is convincing enough, students can not only interact with structures and concepts in ways that expand their 3D reasoning, but they can begin to practice clinical skills, such as assessment sequences and problem solving, all at their own pace [17–20]. One of the key advantages to using VR is that a vast variety of structures or scenarios can be generated with more ease than any dissection or didactic lecture, and be explored and repeated as a student wishes. Additionally, VR applications involving 3D models of anatomical structures are often generated using medical imaging scans, which can account for a plethora of variances, structures [17–19; 21].

VR may have many advantages, but it still only immerses users in a completely alternate ‘reality.’ Some have argued that difficulties bridging the gap between VR and real world applications may temper the benefits [17–19]. But what happens when virtual and digital aspects are layered over real-time reality, if real world images and scenarios could be *augmented* with digital features and objects?

Augmented reality (AR) applications seek to do exactly this—to enhance real world experience with virtual aspects [22,23]. This can be done in a variety of ways, but the most prominent style of AR involves scanning real-time images of the world from a device camera and displaying those images overlaid with digital elements, such as information, 2D graphics, and 3D models that the user can interact with [22–24]. These digital components bear significance on whatever is scanned, such as providing information, showing relevant aspects, or in some cases making a sort of game out of the real-world scenario. AR has recently been used in many capacities involving education, training, simulations, and even in enhancing surgical procedures [18–20; 22–26].

Therefore, the purpose of this study was to harness new technologies but develop it in a very unique manner. We wanted to take advantage of many 3D modelling techniques, and the benefits of ubiquitous digital learning, by attempting to create an effective, novel modality for veterinary students to learn 3D canine head anatomy using highly accurate models generated from MRI and CT scans in an engaging augmented reality (AR) format. Since the canine skull and brain represent some of the more complicated areas for veterinary students to study, and are currently under-represented in available resources, it also provides a unique opportunity to trial new educational approaches. Therefore, the goal of this project was to explore methodologies for segmenting MRI and CT scans, generating and refining models of key elements of the canine skull and brain from them, and making them available in an interactive, intuitive AR platform.

## Materials

### Data

A variety of software and hardware was used in this study to process the data, generate 3D models and integrate them into the final AR application. The following details each medical scan used, software package utilized and the apparatus needed to execute each stage of the study, and is summarised in Tables 1 and 2. This study was considered as sub-threshold for specific ethical approval by the convenor of the School of Veterinary Medicine ethics committee, as the work involved only analysis of data routinely recorded from normal and necessary clinical procedures.

**Table 1:** This demonstrates the scans used for data extraction either from computerised tomography (CT) or magnetic resonance imaging (MRI).

Scan Type	Specifications	Purpose
Canine Head CT	CT images of the head were obtained using a dual slice CT scanner (Siemens Somatom Spirit).	View and segment canine skull
Canine MRI in T1W, T2W, T1W with GAD contrast	Magnetic resonance (MR) imaging of the brain performed using a 1.5-Tesla unit (Siemens Magnetom Essenza)	View and segment canine brain and substructures
Canine T2 CISS MRI (isotropic voxels)	Magnetic resonance (MR) imaging of the brain performed using a 1.5-Tesla unit (Siemens Magnetom Essenza)	View and segment canine brain and substructures

**Table 2:** Software packages used, the reasons for this, and the web links.

Software	Purpose	Web link
3D Slicer	View medical scan data Segment anatomical contours Generate 3D representations and models	https://www.slicer.org/
Autodesk 3ds Max	Create and manually alter polygonal meshes Retopologize existing models	http://www.autodesk.co.uk/products/3ds-max/overview
Zbrush 4R6	Sculpt polygonal meshes with variety of tools Reduce polygon counts with Remesher	http://pixologic.com/zbrush/features/overview/
Unity 5.3	Build interactive applications	https://unity3d.com/
Vuforia	Use plugin in Unity to create Augmented Reality applications using image targets	http://www.vuforia.com/

In addition, the following hardware was used in this study:

- PC (HP Z230 Workstation)
- HTC One M8 mobile phone
- Samsung Tablet Galaxy 10”

## Methods

**Fig 1:**
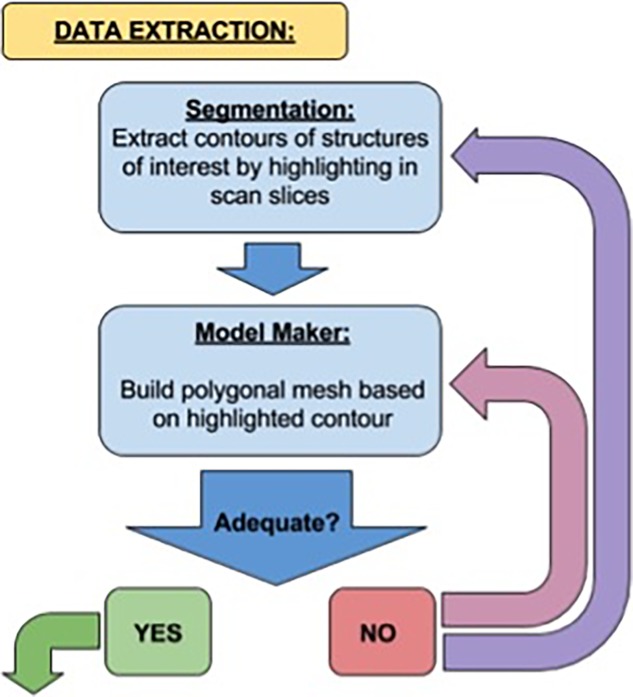
Workflow methodology related to data extraction. From “YES” this leads to Fig 2, Modelling.

**Fig 2:**
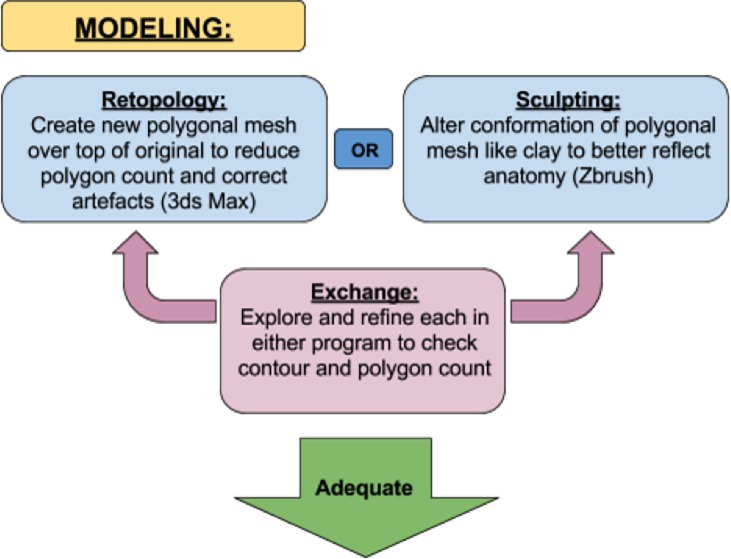
Workflow methodology related to modelling. From “Adequate” it leads to Fig 3, AR Interface.

**Fig 3:**
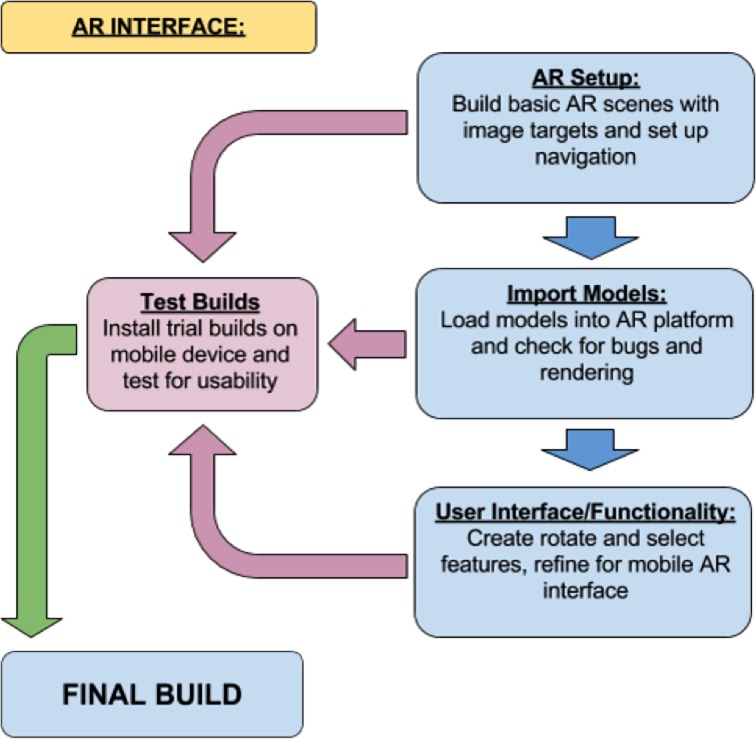
Workflow methodology related to the augmented reality (AR) Interface.

The methodology utilized in this project involved three stages: data extraction, development of accurate 3D anatomical models, and integration of those models into an interactive AR platform. The data extraction process entailed segmentation of the acquired CT and MRI scans of the canine head in 3D Slicer to highlight structures of interest and generate basic models. These models were then refined using a variety of methods in both 3DS Max and Zbrush. Finally, an interactive AR platform was built using Unity, in which a user can interact with each model set in an exciting AR experience. The workflow methodology is summarised in Figs 1–3 and explained below.

### Data extraction

To obtain anatomically accurate models, segmentation was performed on both CT and MRI scans, to reconstruct the canine skull and brain in 3D. Segmentation was performed in 3D Slicer, via a Digital Imaging and Communications in Medicine (DICOM) stack. Manual slice-by-slice segmentation was used with the Threshold Paint to ensure an accurate reconstruction.

The Model Maker in 3D slicer was used to create the 3D skull with a balancing between smoothing of natural ridges and the thinness of the slices used. The Laplacian smoother set to 63 and the Decimation at 0.11 were deemed most appropriate.

**Fig 4:**
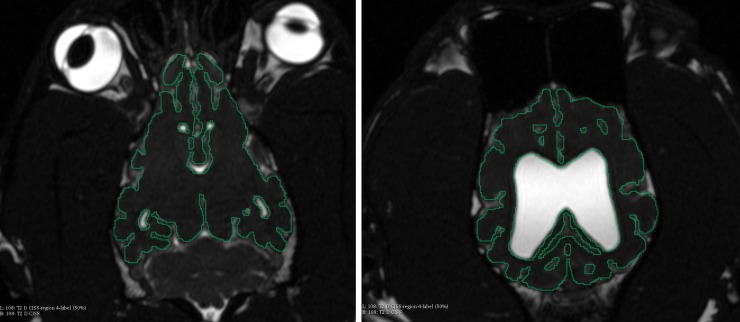
Manual segmentation of the contour of the forebrain in the dorsal plane of the T2 CISS MRI.

The MRI dataset for extraction of the brain had the best resolution in the T2 dorsal plane for segmentation of the brain. To ensure accurate representation of the brain, manual slice-by-slice segmentation was used to identify the larger structures like the forebrain, cerebellum and brainstem, but also to differentiate the different sulci and gyri of the brain. However, due to a number of issues related to the resolution of these scans, it was decided that a T2 CISS MRI would be better suited. The latter is a 3D scan of 1mm^3^ voxels. Although it does not always give good differentiation between soft tissues like grey and white matter, it does have a high contrast between cerebrospinal fluid and soft tissue (Fig 4). Laplacian smoothing set to 38 and Decimation at 0.21 generated a satisfactory model with recognisable contouring of sulci, gyri, lobes and fissure and proportionality of the cerebrum. Accurate volume of the cerebellum could be obtained but nor its surface texture nor its lobes could be precisely recreated using the T2 CISS MRI data. The same was true for the brainstem with accurate volume but not sufficient precise data to reconstruct its exact surface.

#### Retopping and refining skull

**Fig 5:**
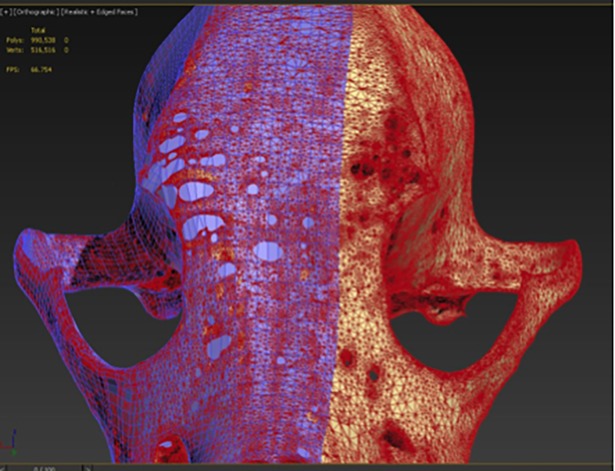
Retopology mesh over half of the original model, with (left) and without edged faces (right).

The skull model generated by 3D Slicer’s Model Maker was smooth, but had many holes. Some of these holes were inherent to the structure of the skull itself, whilst others were artefacts from the scan and shortcomings in the model making. In addition, the skull contained 964,354 polygons, far in excess for rendering in a mobile application. Therefore, manual retopology in 3DS Max was chosen to clean the mesh for mobile applications, yet at the same time remove false holes from the model. “Draw on” was initially selected for the surface of the skull and “conform” was used to gently blanket the plane onto the contour of the skull. However, due to over-sensitivity in this method, a full manual approach to retopology was used. The “strip” and “extend” options were utilised to draw a line of connected square polygons along a contour of the original mesh e.g. along the edge of the mandible, zygomatic arch, midline of the skull and nasal bones. Irregularities were smoothed and regulated using the “Relax” tool, making the spacing between polygons more regular. This was carried out until a clean retopologized mesh was obtained over half of the skull down the midline (Fig 5). As the skull is generally symmetrical, half of the original mesh needed to be refined and retopologized in this manner.

**Fig 6:**
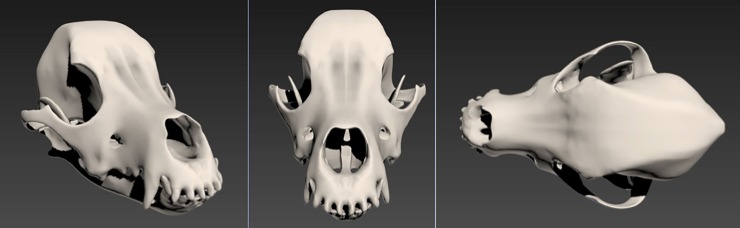
Various views of the completed skull model with the OpenSubdiv modifier and no edged faces.

The “Symmetry” modifier in 2DS Max was utilised to create a symmetrical mirrored mesh, altered and adjusted until the mirrored items lined up, and joined together with the “Bridge” feature of the “Extend” tool. This resulted in a full retoplogized mesh which, in 3D Slicer, had a polygon count of 964,354, but the retopologized skull and mandible had a polygon count of 128,653, 13% of the original count. The final step here was then to use “OpenSubdiv” thus subdividing the existing polygons to smooth the mesh, interpolating the lines, and thus giving a smoothing effect on all edges (Fig 6).

#### Sculpting and remeshing the brain

**Fig 7:**
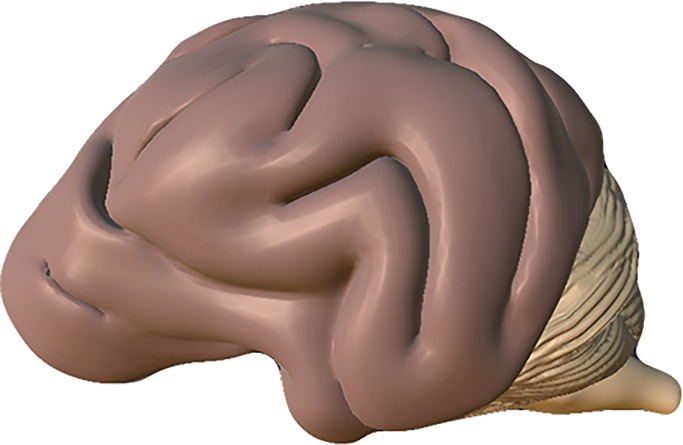
Final brain model when shaded materials have been applied.

To ensure the brain was corrected for minor anomalies, and ensuring the professional appearance to it, Zbrush was used. There were no major issues with the brain, unlike the skull, and following import, a simple smoothing tool was used over each gyrus and elements of the brainstem and cerebellum. The “Dam” tool helped to sharpen the grooves of the sulci, and the “Clay Build-Up” tool was used to thicken areas which had lost mass or developed gaps through the carving process. At this stage, any minor anatomical adjustments could be made ensuring anatomical accuracy of the model. Finally, the brain needed to be retopologized by using the “Remesher” facility in the “Geometry” menu. This allowed for reduction of the polygon count from 706,036 to 342,294 polygons, yet still maintaining a high degree of anatomical accuracy and model cleanliness. The final model once shaded materials were applied can be seen in Fig 7.

### Interface development

#### Trial version

To create an interface, a simplified PC platform was created first with Unity, prior to the augmented reality (AR) platform. This involved creating the basic functionality of three key scenes initially. These were a “Start” screen, a scene for the skull and one for the brain. The “Start” scene included a simple title panel with two buttons, one for the brain and one for the skull. A “Back” button was also included which returned to the opening scene.

Functionality was then added including rotation, highlighting section and user interface (UI) elements linked to sub-object selections. Initially “MouseOrbitZoom” was used as a trial platform to use the camera function to orbit around and zoom in on a selected target during the game. In addition, a Generic Script and a Particular script were used to enable smooth movement between scenes in the interactive application.

Following this trial build for the platform, the AR element was developed using Vuforia development kit for Android in a new Unity project. An AR camera and an Image Target were added to each of the scenes with the updated version of Javascript installed and applied to the HTC One M8 for functionality testing.

#### Final version

As with the experimental trial version, three scenes were created: “Start”, Skull” and “Brain”. The “Start” scene was created using the same AR camera setup as the interactive scenes, with a semi-transparent panel to allow the user to get live camera feed behind the menu. The “Skull” and “Brain” options were adapted from the AR trial scene, this time including a semi-transparent “Top Panel” with title information and a back button for returning to the “Start” menu.

Sagittal sections of the CT skull and MRI brain were used for the final image targets in the Vuforia Developer Portal. Trial builds were then created to test navigation, model placement and model rendering. Test Game Objects were created to trial the functionality from the PC version for the AR. Generic and Particular scripts were imported from the previous trial project, and utilised on trial objects within the AR scene with colours and semi-transparent custom panels corresponding to each object.

#### Functional development

This was created in an alpha version using the final models with colliders. Information panels were created for each sub-object in each scene, with different colours for better visual differentiation of items. A scrollbar was also installed that would work on touch, and scroll freely. Information panels giving a brief description of the sub-object, highlighting important landmarks and features, and links to further discussion and resources were applied to each panel as appropriate.

Simple sphere and capsule colliders were created for each sub-object and Generic and Particular classes were applied to Empty Game Objects and sub-objects, as trialled initially. In addition, rotation functionality needed to be embedded into the AR application. Trialling demonstrated numerous issues: bugs, few were “clamped”, and inability to adjust the rotation for a moving camera. Therefore, a custom-made rotation facility was created. This was designed to be applied directly to the object needing rotation, so that no camera was involved. Conditional statements were applied that prevented rotation on a simple touch only. Rotation only occurred when there was a change in position on the touch or a “delta position”.

For rotation, clamping was trialled, with the notion that simply adjusting the rotation direction based on the object’s orientation would be enough to establish completely intuitive rotation. While this concept proved worthy when the mobile device was held directly in front of the image target, it failed to adjust this performance to odd angles between the image target and the mobile device, as is necessary for a full AR experience.

So instead of clamping the movement, the rotation script was made to retrieve information about the Main Camera’s relative axis, and then to adjust the way the rotation of the object behaves based on these vectors and its local axis. In this manner, the rotation behaviour would always feel intuitive and behave as expected, no matter the angle at which the mobile device is being held around the target.

Once all these elements had been refined, the overall aesthetic of the application was regulated and beautified, to maximize the user’s intuitive feel and enjoyment of the application, while optimizing the potential educational output by ensuring clean, attractive, informative simplicity.

## Results

**Fig 8:**
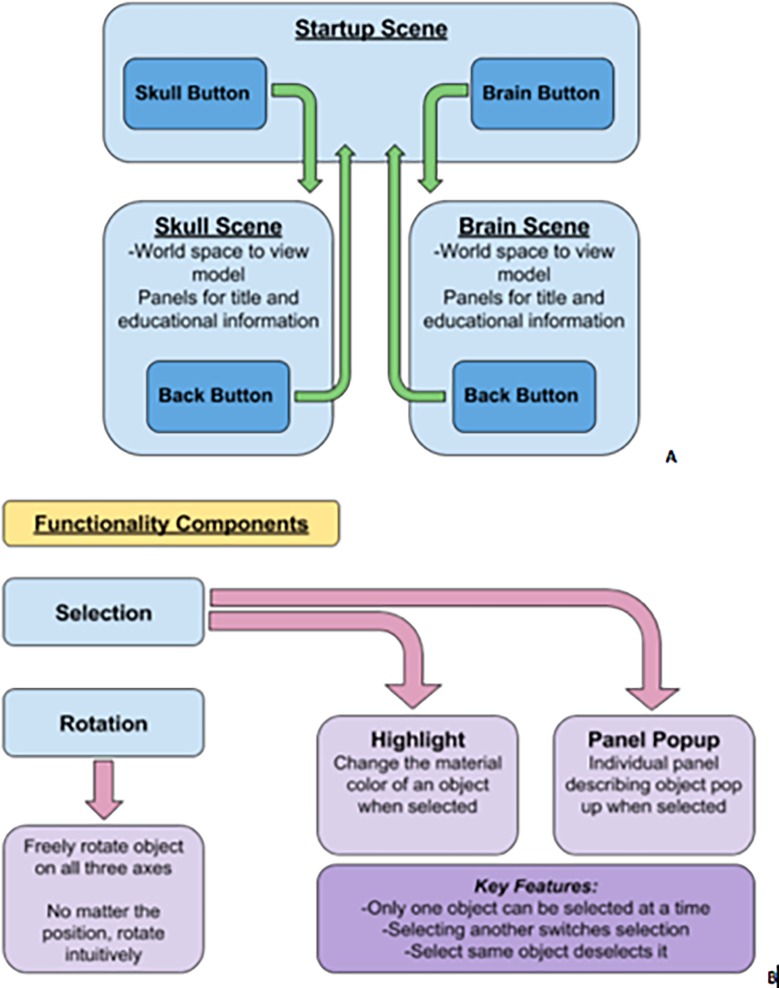
**A.** Basic navigation between scenes. **B.** Intended functionality features.

The methods employed in this study produced the following augmented reality application which allows the user to explore and interact with anatomical models of the canine skull and brain, utilising the functionality depicted in Fig 8A and 8B.

**Fig 9:**
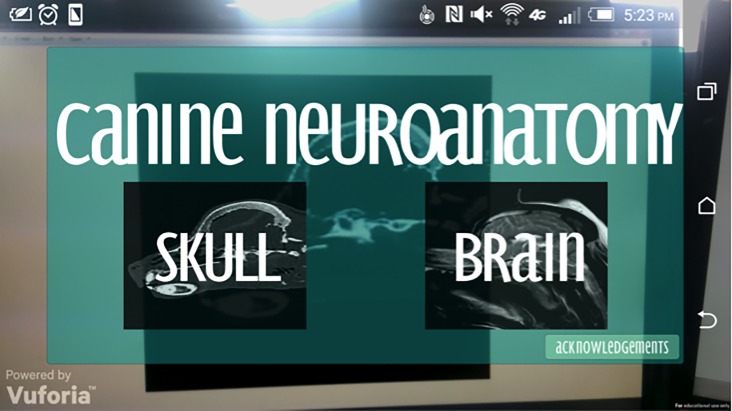
The Start Up menu showing skull, brain and acknowledgments options.

The “Start Up” screen employs a semi-transparent menu panel through which the user gets live camera feed. The buttons included navigate to the following scenes, for the skull, brain or acknowledgements page. The image targets needed for each scene have been rendered on to each button (Fig 9).

**Fig 10:**
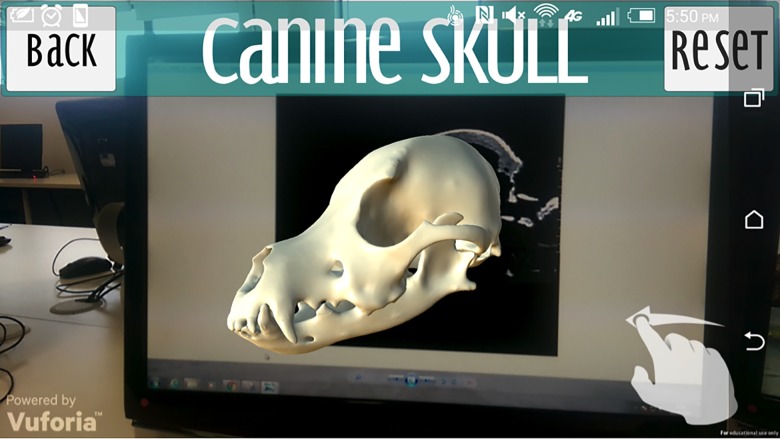
Canine skull menu.

**Fig 11:**
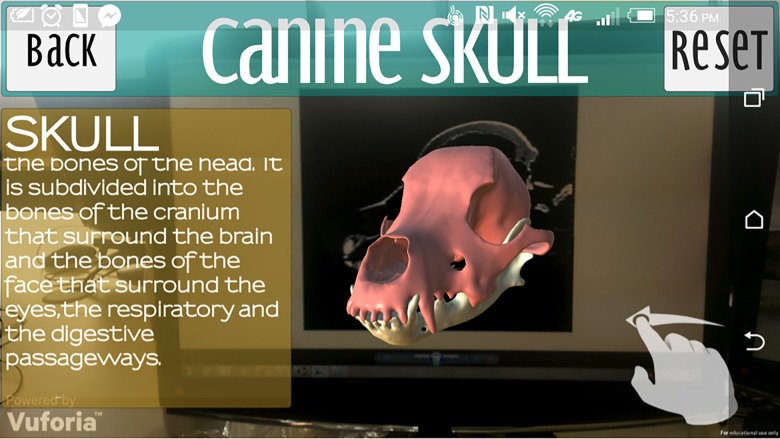
Skull selected in the skull scene.

The “Skull” button navigates to the skull scene where the user hovers over the image target created from the canine CT. This allows the skull model to be visualised as depicted din Fig 10. The user can rotate the model in all three axes by touching and dragging on the screen. The “Reset” button returns the model to its original position. Selecting the main part of the skull highlights it and triggers a pop up panel providing key anatomical information, which can be scrolled through (Fig 11). Selecting individual anatomical territories will reveal further information related to that site.

**Fig 12:**
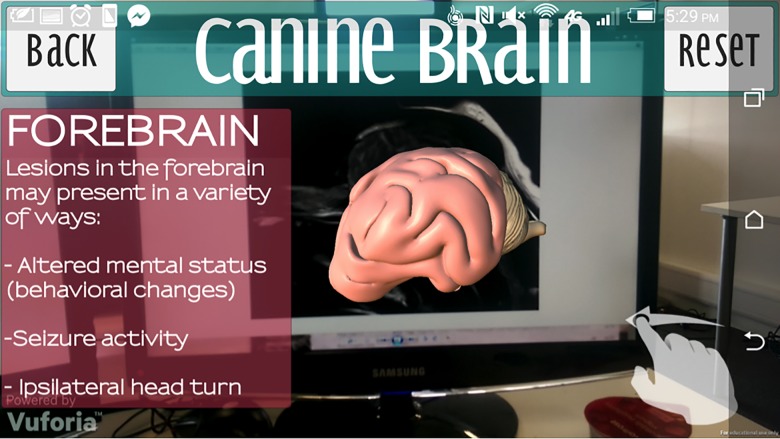
Forebrain selected in the brain scene.

From the “Start Up” menu, the user can also select the “Brain” button, navigating to that topic. The user then utilises the image target, created from the canine MRI. This allows the canine brain model to appear, which the user can rotate and reset in the same fashion as the skull. Selection of specific anatomical regions (e.g. forebrain, cerebellum, brainstem etc.) will then reveal further information (Fig 12). In addition, an acknowledgments page was also created.

## Discussion

The aim of this project was to create a workflow methodology for development of a mobile augmented reality application, potentially to be used for veterinary students learning basic canine head anatomy, both of the skull and brain, in an exciting and intuitive interface. We have shown, through adoption of a variety of commonly available software packages and imaging, how simple it is to create a mobile AR application to potentially be used in future veterinary education.

Anatomy within the veterinary curriculum represents a significant proportion on which clinical training originates, and forms the basis for communication of diagnosis and treatment to the owners and other professionals alike [27,28]. Indeed, teaching within a modern day veterinary curriculum can include a number of modalities, similar to a medical degree programme [29–31]. However, like any curriculum, there are always areas that students find more challenging than other.

Students undertaking veterinary education, like medical training programmes, find the concept of the nervous system, and all related aspects of it, difficult [29,32]. Nowadays, there are a plethora of digital technologies available to aid learning in the medical anatomical field [7–10], but perhaps not so much within the veterinary educational arena [33–35]. Some are emerging; however, they are not advancing at the rate that they are within human anatomical education and training.

Therefore, it is timely that we have developed a clear methodology for the creation of digital veterinary education related products. Given the inadequacies around the teaching of veterinary neuroanatomy, and issues related to visualising structures, modern alternatives are much needed. Indeed, some of the first work in this area was related to computer assisted learning and the development of learning modules via digital lectures, online tutorials and question and answer packages [34]. Certainly this was innovative for its time but technology and our understanding of its educational uses has improved significantly.

Previously the “Visible Animal Project” represented the first 3D anatomical animal model designed specifically for veterinary training. However, the fine detail of canine anatomy was not realised, and lacked detail. From then, “Virtual Canine Anatomy: The Head” was designed and implemented into the first year of the veterinary dissection curriculum within Colorado State University. However, it was built upon 2D views and the illusion was portrayed of a 3D object, but was not as engaging as anticipated.

Thus far, to our knowledge, there is no interactive canine computer aided learning package that offers interactivity and immersion, hence this study using modern day technologies. To enable this, we followed the advice of Clark and Mayer [36] who discussed that for digital technologies to be effective, they advocate the use of good visuals, text and segmenting the different aspects of learning. Within this AR workflow, we have adopted these elements into the canine neuroanatomy to engage the learner in the visualising the detailed anatomy using accessible technology, in this case with a popular smart phone. Indeed, it also complies with it being an inviting and interesting environment, responsive (in that it is visually active) and provides feedback and information related to each of the anatomical areas [37]. Therefore, what we have created has the potential to be engaging in the learning process, however the next stage for this study would be formal testing of the end-user–veterinary students.

## Limitations

In relation to the CT scan data, the only slight drawback was in the fact that it had to be manually manipulated for segmentation. Although it can take a little longer than more automatic techniques, it certainly does allow for identification of clear distinctions between bony structures.

However, the initial MRI dataset that was initially to be used did not have such accessibility and ease of use. The resolution in the T2 dorsal plane showed excellent definition, not only of the larger structures (e.g. cerebrum and cerebellum), but the “out-of-plane” resolution was problematic. The original plan was to segment both larger structures and also the areas between grey and white matter. This would have benefited from an educational perspective in being able to show these clinically relevant areas. It also logically seemed possible under the T1 weighting for grey versus white matter distinction. However, segmenting the full 3D brain and its components produced a cubic and completely unrecognisable model. These scans are not recommended for attempting indirect volume rendering, or the generation of 3D polygonal meshes to be used in other formats. While employing direct volume rendering modules onto the voxel stack itself is able to provide fascinating insight (into a 3D understanding of internal structures to the user), which can be done in both 3D Slicer and Osirix, generation of clean polygonal meshes representing many structures is not feasible. Clinical MRI dataset are therefore of value for an initial assessment of a technique like the one described here to give the student an appropriate and accurate volume relationship between forebrain, brainstem and cerebellum. However, better quality scans (research scan dataset) would be required if more detailed neuroanatomy is needed.

## Future work

Existing models in this field are rather rudimentary and serve to illuminate what is possible, rather than creating a full educational function. However, with careful refinement and investment in these models, there is much opportunity for advancement of the anatomical accuracy through, for example, fine detail of the smaller facial bones. These could be further developed with micro-CT data, to ensure a more accurate representation of the skeletal anatomy.

A difficulty with this type of work, which merits further research, is refining the accuracy and detail of the canine brain for a mobile augmented reality application. The potential here is for higher resolution MR datasets, dissections and photogrammetry combined to provide a photorealistic and highly accurate reconstruction.

This full incorporation of anatomical and also potentially neuroanatomy and neuroscience would need to be educationally validated by those veterinary surgeons who specialise in neurosurgery and clinically applied research. It would also need validation from ultimately the end user–students. A well-designed trial with both alpha and beta phase testing would be necessary to ensure the application of this type of teaching tool.

## Conclusion

The purpose of this project was to establish the processes for a methodology in the creation of an augmented reality application for basic canine head anatomy. We have clearly identified the advantages and drawbacks of different approaches in the creation of a robust and interactive augmented reality tool for veterinary education. Of course, further validation is needed both from specialist neurological and neurosurgical veterinary clinicians and the students themselves. However, this process clearly sets out the workflow methodology in the creation of a novel, innovative, different and cutting edge tool for enhancing learning opportunities with a visual, tactile and engaging manner. Now, we have shown the basic recipe for those involved in veterinary education who are keen to develop ideas and innovations, but tailor make it for each of your teaching, learning and assessment methods both locally, nationally and internationally. This type of technological advance and application is not only limited to veterinary education but can be opened up to ensure an immersive learning environment for anything requiring visual and tactile learning.

## References

[1] Sugand K, Abrahams P, Khurana A. The Anatomy of Anatomy: A Review for its Modernisation. Anatomical Sciences Education. 2010; 3(2): 83–93. doi: 10.1002/ase.139 20205265 10.1002/ase.139

[2] Elizondo‐Omaña R.E., Guzmán‐López S. and De Los Angeles García‐Rodríguez M. Dissection as a teaching tool: past, present, and future. The Anatomical Record Part B: The New Anatomist. 2005; 285(1): 11–15. 10.1002/ar.b.20070 16032753

[3] Turney B.W. Anatomy in a modern medical curriculum. Ann R Coll Surg Engl. 2007; 89: 104–107. doi: 10.1308/003588407X168244 17346399 10.1308/003588407X168244 PMC1964553

[4] Verhoeven BH, Verwijnen GM, Scherpbier AJ, Van Der Vleuten CPM. Growth of medical knowledge. Med Educ. 2002; 36: 711–717. 12191053 10.1046/j.1365-2923.2002.01268.x

[5] Patel KM, Moxham BJ. The relationships between learning outcomes and methods of teaching anatomy as perceived by professional anatomists. Clinical Anatomy. 2008; 21: 182–189. doi: 10.1002/ca.20584 18189277 10.1002/ca.20584

[6] Ashwell KW, Halasz P. An Acrobat-based program for gross anatomy revision. Medical Education. 2004; 38: 1185–1186. doi: 10.1111/j.1365-2929.2004.01990.x 15507017 10.1111/j.1365-2929.2004.01990.x

[7] 3D4Medical. http://www.3d4medical.com [Accessed 11th July 2017]

[8] Anatomy.TV https://www.anatomy.tv [Accessed 11th July 2017]

[9] BodyViz http://www.bodyviz.com [Accessed 11th July 2017]

[10] Cyber Anatomy HolographicTM http://cyber-anatomy.com/Holographic.php [Accessed 11th July 2017]

[11] Spitzer VM, Whitlock DG. Atlas of the Visible Human Male: Reverse Engineering of the Human Body. 1998 Sadbury, Jones & Barlett.

[12] Robertson D, Johnston W, Nip W. The Whole Frog Project. The Second International WWW Conference 1994 Available: http://froggy.lbl.gov/papers/WWW.94/paper.html [Accessed 11^th^ July 2017]

[13] Toga AW, Santori EM, Hazani R, Ambach K. A 3D digital map of the rat brain. Brain Research Bulletin. 1995; 38(1): 77–85. 7552378 10.1016/0361-9230(95)00074-o

[14] Martinelli MJ, Kuriashkin IV, Carragher BO, Clarkson RB, Baker GJ. Magnetic Resonance Imaging of the Equine Metacarpophalangeal Joint: Three Dimensional Reconstruction and Anatomic Analysis. Veterinary Radiology and Ultrasound. 1997; 38(3): 193–199. 9238790 10.1111/j.1740-8261.1997.tb00840.x

[15] Bottcher P, Maierl J, Schiemann T, Glaser C, Weller R, Hoehne KH et al The Visible Animal Project: A Three Dimensional, Digital Database for High Quality Three Dimensional Reconstruction. Veterinary Radiology and Ultrasound. 1999; 40(6): 611–616. 10608688 10.1111/j.1740-8261.1999.tb00887.x

[16] Burdea GC, Coiffet P. Virtual Reality Technology, second edition 2003 Wiley–Interscience.

[17] Chien CH, Chen CH, Jeng TS. 2010 3 An interactive augmented reality system for learning anatomy structure In Proceedings of the International MultiConference of Engineers and Computer Scientists (Vol. 1). International Association of Engineers

[18] Ma M, Fallavollita P, Seelbach I, Heide AM, Euler E, Waschke J et al Personalized augmented reality for anatomy education. Clinical Anatomy. 2016; 29: 446–453. doi: 10.1002/ca.22675 26646315 10.1002/ca.22675

[19] Zhu E., Hadadgar A., Masiello I. and Zary N., 2014 Augmented reality in healthcare education: an integrative review. PeerJ, 2, p.e469 doi: 10.7717/peerj.469 25071992 10.7717/peerj.469 PMC4103088

[20] Seymour NE, Gallagher AG, Roman SA, O’Brien MK, Bansal VK, Andersen D.K. et al Virtual reality training improves operating room performance: results of a randomized, double-blinded study. Annals of Surgery. 2002; 236(4): 458–464. doi: 10.1097/01.SLA.0000028969.51489.B4 12368674 10.1097/00000658-200210000-00008 PMC1422600

[21] Khot Z, Quinlan K, Norman GR, Wainman B. The relative effectiveness of computer‐based and traditional resources for education in anatomy. Anatomical Sciences Education. 2013; 6(4), pp.211–215 doi: 10.1002/ase.1355 23509000 10.1002/ase.1355

[22] Barsom EZ, Graafland M, Schijven MP. Systematic review on the effectiveness of augmented reality applications in medical training. Surgical Endoscopy. 2016; 30(10): 4174–4183. doi: 10.1007/s00464-016-4800-6 26905573 10.1007/s00464-016-4800-6 PMC5009168

[23] Jamali SS, Shiratuddin MF, Wong KW, Oskam CL. Utilising Mobile-Augmented Reality for Learning Human Anatomy. Procedia-Social and Behavioral Sciences. 2015; 197: 659–668.

[24] Kamphuis C, Barsom E, Schijven M, Christoph N. Augmented reality in medical education? Perspectives on Medical Education. 2014; 3(4): 300–311. doi: 10.1007/s40037-013-0107-7 24464832 10.1007/s40037-013-0107-7 PMC4152469

[25] Baker DK, Fryberger CT, Ponce BA. The Emergence of Augmented Reality in Orthopaedic Surgery and Education. The Orthopaedic Journal at Harvard Medical School; 2015: 8–16.

[26] Parkes R, Forrest N, Baillie S. A mixed reality simulator for feline abdominal palpation training in veterinary medicine. Studies in Health Technology and Informatics. 2009; 142:244–246. 19377159

[27] Boon JM, Meiring JH, Richards PA. Clinical Anatomy as the Basis for Clinical Examination: Development and Evaluation of an Introduction to Clinical Examination in a Problem-Orientated Medical Curriculum. Clinical Anatomy. 2002; 15: 45–50. doi: 10.1002/ca.1091 11835544 10.1002/ca.1091

[28] Turney BW. Anatomy in a Modern Medical Curriculum. Annals of the Royal College of Surgeons of England. 2007; 89(2): 102–107. 10.1308/003588407X168244 PMC1964553 17346399

[29] Jastrow H, Hollinderbaumer A. On the Use and Value of New Media and How Medical Students Assess Their Effectiveness in Learning Anatomy. The Anatomical Record: Part B, New Anatomist. 2004; 280(1): 20–29. 10.1002/ar.b.20027 15382114

[30] Raffan H, Guevar J, Poyade M, Rea PM (2017) Canine neuroanatomy: Development of a 3D reconstruction and interactive application for undergraduate veterinary education. PLoS ONE 12(2): e0168911 https://doi.org/10.1371/journal.pone.0168911 doi: 10.1371/journal.pone.0168911 28192461 10.1371/journal.pone.0168911 PMC5305238

[31] Dale V. Educational methods and technologies in undergraduate veterinary medicine. A case study of veterinary teaching and learning at Glasgow, 1949–2006. Thesis submitted for the degree of Doctor of Philosophy in The Faculty of Veterinary Medicine, University of Glasgow, 2008.

[32] Ramos RL, Smith PT, Croll SD, Brumberg JC. Demonstrating Cerebral Vascular Networks: A Comparison of Methods for the Teaching Laboratory. Journal of Undergraduate Neuroscience Education. 2008; 6(2): 53–59. PMC3592665 23493521

[33] Borden NM. 3D Angiographic Atlas of Neurovascular Anatomy and Pathology, New York: Cambridge University Press 2007.

[34] Holmes MA, Nicholls PK. Computer-aided veterinary learning at the University of Cambridge. The Veterinary Record. 1996; 138(9): 199–203. 8686151 10.1136/vr.138.9.199

[35] Linton A, Schoenfeld-Tacher R, Whalen LR. Developing and Implementing an Assesment Method to Evaluate a Virtual Canine Anatomy Program. Journal of Veterinary Medical Education. 2005; 32(2): 249–254. 16078179 10.3138/jvme.32.2.249

[36] Clark RC and Mayer RE. e-Learning and the Science of Instruction: Proven Guidelines for Consumers and Designers of Multimedia Learning, Third Edition 2011 John Wiley & Sons, Inc. 10.1002/9781118255971.

[37] Mayer RE. Computer Games for Learning: An Evidence-Based Approach Hardcover – 8 MIT Press 2014. ISBN-10: 0262027577.

